# Impact of Dual-Tracer ⁶⁸Ga-PSMA/¹⁸F-FDG PET/CT on the Characterization of Tumor Heterogeneity and Clinical Decision-Making in Metastatic Prostate Cancer: An Index Case From Ecuador

**DOI:** 10.7759/cureus.105612

**Published:** 2026-03-21

**Authors:** Sebastian Molina, Adriana P Noboa, Cynthia Lopez, Francisco Cornejo

**Affiliations:** 1 Unidad PET Scan, Hospital Metropolitano, Quito, ECU; 2 Oncology, Hospital Metropolitano, Quito, ECU; 3 Urology, Hospital Metropolitano, Quito, ECU

**Keywords:** dual pet/ct, fdg, fluorine-18, gallium-68, molecular and oncologic imaging, prostate cancer, psma, tumor heterogeneity

## Abstract

Prostate cancer is one of the most common malignancies among men worldwide, and its incidence is also significant in Ecuador. This disease can present marked biological heterogeneity, particularly in advanced stages, such as metastatic and castration-resistant prostate cancer, which may influence both imaging findings and therapeutic decisions. We report an index case in Ecuador in which combined gallium-68 prostate-specific membrane antigen (^68^Ga-PSMA) and fluorine-18 fluorodeoxyglucose (^18^F-FDG) positron emission tomography/computed tomography (PET/CT) was used for the comprehensive characterization and longitudinal follow-up of a patient with high-volume metastatic prostate cancer, highlighting the complementary metabolic and molecular information provided by both imaging tracers. The dual-tracer approach revealed discordant lesions - with differential PSMA and FDG uptake - highlighting substantial metabolic heterogeneity and guiding an intensified therapeutic strategy consisting of combined chemotherapy (docetaxel + carboplatin), androgen deprivation therapy, and androgen receptor inhibition. Follow-up dual PET imaging demonstrated a 99% reduction in tumor burden and complete metabolic remission, underscoring the value of this approach in treatment planning, assessment of disease extent, and response evaluation. This case illustrates the importance of dual-tracer PET/CT with PSMA and FDG as an essential tool for accurate disease characterization, personalized treatment selection, and identification of aggressive tumor subpopulations in advanced prostate cancer.

## Introduction

Prostate cancer is one of the most common malignant neoplasms in men and remains a major cause of cancer-related mortality worldwide [1]. According to the Global Cancer Observatory, prostate cancer is the most frequently diagnosed cancer among men in Ecuador, with an incidence of 24.9% in 2022 [2].

Despite advances in diagnostic and therapeutic strategies, 30-40% of patients experience recurrence or metastatic progression after initial treatment [3]. This underscores the importance of accurately characterizing all metastatic lesions to guide optimal therapy and improve patient prognosis.

PET using radiotracers targeting the prostate-specific membrane antigen (PSMA), such as gallium-68 PSMA (^68^Ga-PSMA), is an established imaging modality for detecting disease recurrence and evaluating tumor extent, particularly in the setting of biochemical relapse [4]. However, in advanced disease with significant tumor heterogeneity, certain metastatic lesions may lack PSMA overexpression and therefore remain undetected on ^68^Ga-PSMA PET/CT. Instead, these lesions may demonstrate high glycolytic activity, which is identifiable using fluorine-18 fluorodeoxyglucose (^18^F-FDG) PET/CT [5,6].

Recent studies have shown that patients with castration-resistant prostate cancer may exhibit discordant lesions characterized by positive ^18^F-FDG uptake and absent ^68^Ga-PSMA uptake, reflecting heterogeneous PSMA expression. This pattern has been associated with higher prostate-specific antigen (PSA) levels, more aggressive histologic subtypes (e.g., intraductal carcinoma and neuroendocrine differentiation), and poorer overall prognosis [7]. However, it is important to note that PSA may remain low in dedifferentiated tumors, as illustrated in this case, where extensive PSMA-avid and FDG-avid disease was present on PET imaging despite a PSA level of <10 ng/mL [8]. Therefore, PSA alone is insufficient for assessing tumor aggressiveness, reinforcing the added value of ^68^Ga-PSMA and ^18^F-FDG dual-tracer PET for comprehensive staging, which provides clinically meaningful information for therapeutic selection - particularly in patients being considered for PSMA-targeted theranostic therapy [7,8].

In patients with high-volume metastatic castration-sensitive prostate cancer (mCSPC), systemic treatment intensification has demonstrated significant clinical benefits. Combination therapy with androgen deprivation therapy (ADT), docetaxel, and a next-generation androgen receptor pathway inhibitor (ARPI) - such as abiraterone, enzalutamide, or darolutamide - has been shown to improve overall survival (OS) and delay disease progression compared with ADT alone or ADT plus docetaxel [9].

The PEACE-1 trial demonstrated that adding abiraterone to ADT + docetaxel in patients with mCSPC, particularly those with high-volume disease, resulted in a significant improvement in OS (median: 5.1 years vs. 4.4 years; HR: 0.75) and radiographic progression-free survival (PFS) [9].

Similarly, the Androgen Receptor Antagonist in Combination with Standard Therapy for Metastatic Hormone-Sensitive Prostate Cancer (ARASENS) trial showed that incorporating darolutamide into ADT + docetaxel reduced the risk of death by 32.5% compared with ADT + docetaxel + placebo (HR: 0.68), without a significant increase in severe adverse events [10].

The Enzalutamide in Metastatic Hormone-Sensitive Prostate Cancer (ENZAMET) study further supported the benefit of adding enzalutamide to ADT (with or without docetaxel), demonstrating improved PFS and OS, especially among patients with high tumor burden [11].

These findings have led major international guidelines - including National Comprehensive Cancer Network (NCCN), European Urology Oncology (EAU), and American Urological Association (AUA) - to recommend considering treatment intensification in patients with high-volume mCSPC. Targeting multiple tumor signaling pathways early in the disease course allows for more effective disease control, delayed development of hormonal resistance, and improved OS and quality of life [3,12].

The combination of docetaxel and carboplatin has also been explored as an intensified therapeutic strategy in mCSPC, particularly in patients with aggressive features, such as high tumor burden, cellular dedifferentiation, or deoxyribonucleic acid (DNA) repair pathway mutations [13]. Among patients with castration-resistant prostate cancer who were refractory to docetaxel, weekly docetaxel (35 mg/m² on days 1, 8, and 15 of a 28-day cycle) combined with carboplatin (area under the curve (AUC): 5 on day one) achieved PSA reductions ≥50% in approximately 51% of patients, with median PFS of 6.5 months and OS of 15.8 months, although with significant but reversible hematologic toxicity [13].

Similarly, the phase II study by Ross et al. evaluating the same combination in men with metastatic castration-resistant disease following docetaxel treatment reported PSA responses ≥50% in approximately 18% of patients, with a median PFS of three months and OS of 12.4 months. Notably, patients with prior response to docetaxel tended to have better outcomes with this regimen [14]. A more recent trial combining docetaxel, carboplatin, and estramustine in castration-resistant disease demonstrated PSA reductions >30% in 18 of 20 patients and >50% in 14 patients, with a median OS of 11 months and PFS of approximately 6.5 months [15].

Importantly, among patients harboring homologous recombination repair gene mutations, such as breast cancer gene 1 (*BRCA1*), *BRCA2*, or partner and localizer of *BRCA2* (*PALB2*), the docetaxel-carboplatin combination appears particularly effective [16]. In the study by Smith et al., approximately 69% of patients with these mutations achieved PSA reductions ≥50%, with response rates up to 78% in those with *BRCA* mutations, median PFS of 8.4 months, and median OS of approximately two years [10]. Grade 3 toxicities - including thrombocytopenia and anemia - were common, and one case of grade 4 toxicity was reported [16].

The observed benefits of this combination include significant PSA responses, prolonged survival, delayed disease progression, and symptomatic improvement, such as pain reduction and enhanced quality of life in selected patients. However, its use is limited by considerable hematologic toxicity - including grade 3-4 neutropenia, anemia, and thrombocytopenia - which may require growth factor support or dose adjustments [13]. Additionally, responses may be modest or short-lived in patients previously treated with docetaxel, and current evidence is largely restricted to castration-resistant disease, with limited data in the castration-sensitive setting [14,15].

## Case presentation

**Figure 1:**
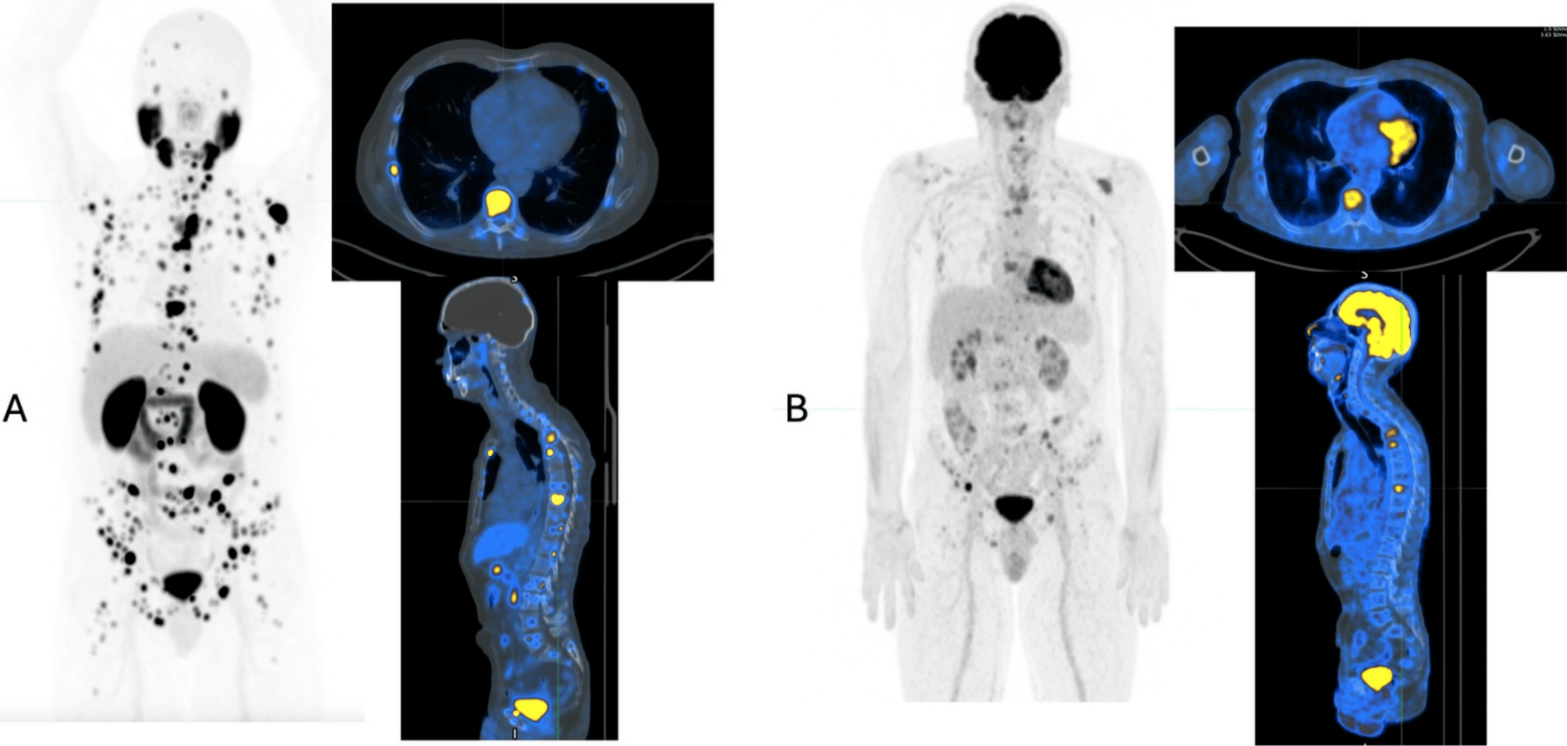
Imaging studies performed at the PET scan unit, Hospital Metropolitano, Quito, Ecuador. (A) ^68^Ga-PSMA PET showing multiple lesions with overexpression of PSMA receptors involving the skeleton and the prostate bed, according to the standardized molecular imaging classification system PROMISE v2 (Prostate Molecular Imaging Standardized Evaluation): mITr, N0, M1b diss [4]. (B) ^18^F-FDG PET demonstrating hypermetabolic osseous lesions disseminated throughout the axial and appendicular skeleton. ^18^F-FDG: fluorine-18 fluorodeoxyglucose; ^68^Ga-PSMA: gallium-68 prostate-specific membrane antigen

A 69-year-old man with a history of acinar adenocarcinoma of the prostate, Gleason score 7 (3+4), diagnosed in 2022 and treated with radical prostatectomy, presented in August 2024 with biochemical recurrence, evidenced by a PSA level of 5.96 ng/mL, accompanied by thoracic and lumbar pain that was exacerbated by postural changes. A ^68^Ga-PSMA PET/CT performed in August 2024 revealed multiple foci of PSMA receptor overexpression within the skeleton and the prostate bed. Additionally, the scan demonstrated multiple mixed lesions suspicious for metastatic disease that lacked PSMA avidity, prompting a recommendation for ^18^F-FDG PET/CT. The subsequent ^18^F-FDG PET/CT performed in September 2024 showed numerous hypermetabolic osseous lesions disseminated throughout the axial and appendicular skeleton, as shown in Figure 1.

Given these findings, the patient underwent palliative radiotherapy consisting of 27 Gray (Gy) directed to the left shoulder, thoracic spine (T4-T10), and the region extending from L3 to the sacroiliac joints, administered between September 23 and 25, 2024. As the patient was classified as having high-volume mCSPC - characterized by extensive osseous metastases and metastatic lymphadenopathy - complete androgen blockade was pursued through bilateral simple orchiectomy in September 2024.

Subsequently, combined chemotherapy with docetaxel (75 mg/m²) and AUC 5 was initiated, with the patient completing six cycles by February 2025. During treatment, he developed reversible hematologic toxicity that required granulocyte colony-stimulating factor support. In parallel, therapy with the next-generation androgen receptor inhibitor darolutamide was initiated at an oral dose of 600 mg every 12 hours. Bone-protective treatment with denosumab 60 mg subcutaneously every six months was also added.

This multidisciplinary approach resulted in adequate control of metastatic disease, providing comprehensive management of the tumor burden while minimizing treatment-related complications.

**Figure 2:**
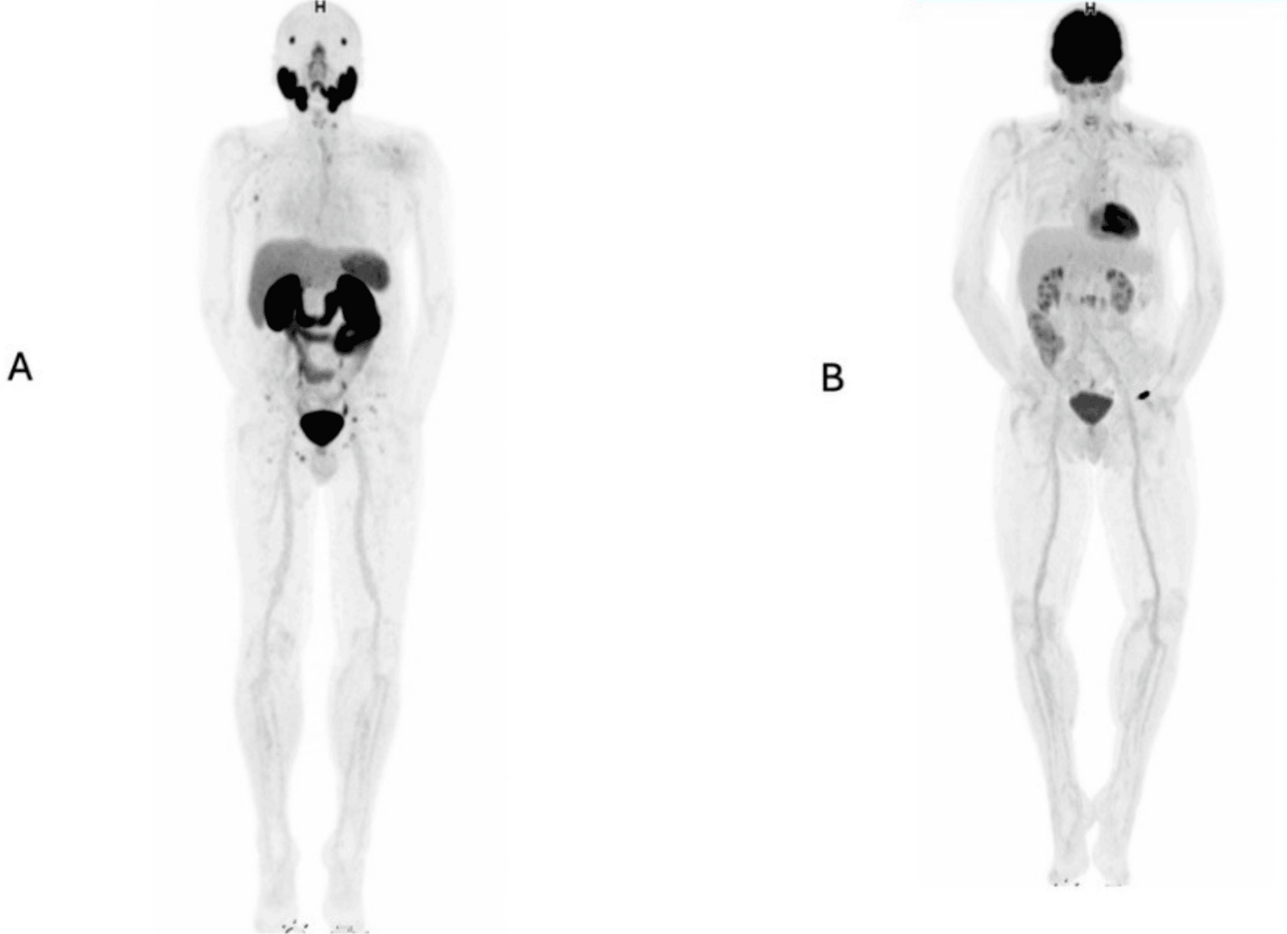
Imaging studies performed at the PET SCAN unit, Hospital Metropolitano, Quito, Ecuador. (A) ^68^Ga-PSMA PET demonstrating a marked metabolic tumor response, quantified using artificial intelligence-based tumor burden analysis: metabolic tumor volume (MTV) decreased from 489 cc to 3.59 cc, and total lesion activity (TLA) decreased from 3960 to 12.9, corresponding to a 99% reduction in tumor volume and a 92% reduction in metabolic activity, according to the standardized molecular imaging classification system PROMISE v2 (Prostate Molecular Imaging Standardized Evaluation): mITr, N0, M1b diss [4]. (B) ^18^F-FDG PET showing complete metabolic response, with no evidence of residual hypermetabolic lesions. A hypermetabolic focus visualized in the left pelvic region corresponds to the radiopharmaceutical injection site at the dorsum of the hand. ^18^F-FDG: fluorine-18 fluorodeoxyglucose; ^68^Ga-PSMA: gallium-68 prostate-specific membrane antigen

On post-treatment follow-up, a ^68^Ga-PSMA PET/CT performed in April 2025 demonstrated a 99% reduction in tumor volume and a 92% reduction in metabolic activity. Given the prior evidence of cellular dedifferentiation, a follow-up ^18^F-FDG PET/CT was performed in July 2025, which showed complete metabolic remission, as shown in Figure 2.

## Discussion

In this clinical case, the relevance of combining ^68^Ga-PSMA and ^18^F-FDG PET/CT for the evaluation of metastatic prostate cancer is highlighted. The complementary role of dual-tracer imaging was particularly relevant in this case. The initial ^68^Ga-PSMA PET/CT demonstrated extensive skeletal involvement but also identified several lesions suspicious for metastatic disease that lacked clear PSMA avidity. This finding raised concern for possible tumor heterogeneity and prompted the subsequent ^18^F-FDG PET/CT examination. The FDG study confirmed widespread hypermetabolic skeletal disease, supporting the presence of biologically aggressive tumor components with potentially lower PSMA expression. The combined interpretation of both imaging modalities provided a more comprehensive characterization of the disease and reinforced the decision to pursue an intensified multimodal therapeutic strategy, including systemic chemotherapy, androgen deprivation therapy, and androgen receptor pathway inhibition. Furthermore, the use of dual-tracer PET imaging during follow-up allowed a more complete evaluation of treatment response, reducing the risk of underestimating residual metabolically active disease [5,6].

Consistently, studies such as that of Mahal et al. have shown that high-grade prostate cancers may exhibit relatively low PSA levels despite substantial tumor burden, underscoring that a modestly elevated PSA value, such as the 5.96 ng/mL observed in this patient, does not exclude extensive metastatic disease nor indicate a biologically indolent phenotype [17].

Dual-tracer PET was also essential for monitoring treatment response during multimodal therapy, preventing underestimation of tumor burden and supporting more accurate therapeutic decision-making. In this case, ^68^Ga-PSMA PET/CT demonstrated a 99% reduction in tumor volume and a 92% decrease in metabolic activity, followed by ^18^F-FDG PET/CT confirming complete metabolic remission [7,8,18].

Mutsuddy et al. provided another clear example of the value of dual-tracer imaging with PSMA and FDG, demonstrating its capacity for comprehensive disease characterization [19]. Studies such as those by Dai et al. have shown that FDG positivity correlates with elevated PSA levels and more aggressive tumor subtypes [5], whereas others, such as Mahal et al., have demonstrated that high-grade tumors with low PSA levels may present with extensive disease - emphasizing that PSA does not always correlate with true tumor burden, particularly in dedifferentiated prostate cancers [17].

Furthermore, the prospective study by Thang et al. showed that, in patients undergoing dual-tracer PET, low PSMA uptake - particularly in castration-resistant prostate cancer - was associated with extremely poor prognosis, with an expected survival of only one to four months. These findings underscore the critical importance of accurate disease characterization when determining treatment strategies and evaluating suitability for PSMA-targeted theranostic therapies [20].

The present case is particularly relevant because it clearly illustrates how the implementation of dual-tracer PET substantially altered the understanding of the patient's tumor biology and redirected management toward an intensified and personalized therapeutic approach, aligned with recommendations derived from contemporary clinical trials and prospective studies [9-15,20]. Reporting this experience is valuable for the Ecuadorian medical community, as it demonstrates the real-world clinical applicability of dual-tracer PET in complex scenarios and highlights its potential to influence therapeutic decision-making and patient prognosis.

## Conclusions

This index case from Ecuador demonstrates the clinical utility of dual-tracer ^68^Ga-PSMA and ^18^F-FDG PET/CT in metastatic prostate cancer. The dual-imaging approach enabled comprehensive characterization of tumor heterogeneity, guided an intensified and personalized therapeutic strategy, and allowed accurate monitoring of treatment response. Follow-up imaging confirmed a near-complete response on ^68^Ga-PSMA and complete metabolic remission on ^18^F-FDG PET/CT, underscoring the value of this modality in assessing disease biology beyond conventional biomarkers.

In parallel, the use of combined docetaxel and carboplatin chemotherapy highlights a potentially effective option in aggressive prostate cancer phenotypes characterized by dedifferentiation, where conventional hormonal therapies may be insufficient. Although current evidence supporting this approach is largely derived from castration-resistant disease and later treatment lines, this case illustrates how dual-tracer PET/CT can inform therapeutic selection, prognostic assessment, and risk stratification. Overall, dual-tracer PET/CT represents a powerful tool for optimizing staging, treatment planning, and follow-up in selected patients with advanced prostate cancer.
